# A Plausible Prebiotic One-Pot Synthesis of Orotate and Pyruvate Suggestive of Common Protometabolic Pathways

**DOI:** 10.1002/anie.202112572

**Published:** 2022-01-27

**Authors:** Alyssa P. Clay, Rachel E. Cooke, Ravi Kumar, Mahipal Yadav, Ramanarayanan Krishnamurthy, Greg Springsteen

**Affiliations:** Department of Chemistry, Furman University, 3300 Poinsett Hwy, Greenville, SC 29613 (USA); NSF-NASA Center for Chemical Evolution, Atlanta, GA 30332 (USA); Department of Chemistry, Furman University, 3300 Poinsett Hwy, Greenville, SC 29613 (USA); NSF-NASA Center for Chemical Evolution, Atlanta, GA 30332 (USA); Department of Chemistry, The Scripps Research Institute, 10550 North Torrey Pines Road, La Jolla, CA 92037 (USA); NSF-NASA Center for Chemical Evolution, Atlanta, GA 30332 (USA); Department of Chemistry, The Scripps Research Institute, 10550 North Torrey Pines Road, La Jolla, CA 92037 (USA); NSF-NASA Center for Chemical Evolution, Atlanta, GA 30332 (USA); Department of Chemistry, The Scripps Research Institute, 10550 North Torrey Pines Road, La Jolla, CA 92037 (USA); NSF-NASA Center for Chemical Evolution, Atlanta, GA 30332 (USA); Department of Chemistry, Furman University, 3300 Poinsett Hwy, Greenville, SC 29613 (USA); NSF-NASA Center for Chemical Evolution, Atlanta, GA 30332 (USA)

**Keywords:** Pyrimidine Synthesis, Pyruvate Synthesis, Prebiotic Chemistry

## Abstract

A reaction between two prebiotically plausible building blocks, hydantoin and glyoxylate, generates both the nucleobase orotate, a precursor of biological pyrimidines, and pyruvate, a core metabolite in the citric acid cycle and amino acid biosynthesis. The reaction proceeds in water to provide significant yields of the two widely divergent chemical motifs. Additionally, the reaction of thiohydantoin and glyoxylate produces thioorotate in high yield under neutral aqueous conditions. The use of an open-chain thiohydantoin derivative also enables the potential pre-positioning of a nucleosidic bond prior to the synthesis of an orotate nucleoside. The observation that diverse building blocks of modern metabolism can be produced in a single reaction pot, from common reactants under mild conditions, supports the plausibility of orthogonal chemistries operating at the origins of chemical evolution.

## Graphical Abstract

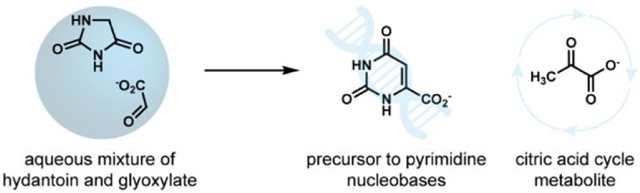

A prebiotic synthesis of the nucleobase orotate, and the citric acid cycle intermediate pyruvate, proceeds in a single pot from two small glycine derivatives, hydantoin and glyoxylate, under mild aqueous conditions. These findings support a co-evolution of pathways to core protometabolites and nucleic acid building blocks in a common environment. Additionally, open-chain thiohydantoin derivatives enable a synthesis of thioorotate nucleosides.

In the quest to understand the chemical origins of life, the RNA-world and metabolism-world hypotheses have often been considered to offer two competing approaches.^[1–3]^ This has led to the impression that one of these systems appeared first and gave rise to the other. However, demonstrations of the formation of components of both worlds via common pathways may begin to lay the groundwork to reconcile the perceived orthogonal differences between them.^[4]^ As a step in that direction, herein we describe a chemistry that is able to generate building blocks of both nucleic acid synthesis and carboxylate metabolism. Orotate, a pyrimidine precursor to uridine and cytidine (Scheme 1), and pyruvate, an α-keto acid linking glycolysis to the citric acid cycle and fatty acid biosynthesis, are produced by the same sequence of reactions (Scheme 2) in water from two simple glycine derivatives, hydantoin and glyoxylate. Such shared pathways and chemistries may help initiate a symbiotic co-evolution of ketoacid-based protometabolic pathways^[5,6]^ concurrent with nucleic acid synthesis.^[7]^

Demonstrations of prebiotically plausible syntheses of multiple components of an early metabolism, under mild conditions, would simplify hypotheses that seek to merge the availability of building blocks with the function of early protobiopolymers. These syntheses would be particularly compelling if they appeared to enable a smooth evolutionary trajectory from abiotic to modern biosynthetic routes. However, current hypotheses for the origins of biologically relevant building blocks often suffer from harsh and/or incompatible conditions differing in fundamental chemistry from their modern biological counterparts. For example, proposed conditions for the prebiotic synthesis of orotate (the modern biosynthetic precursor to pyrimidine nucleotides from aspartate, Scheme 1), have included: 1) the acid hydrolysis of HCN polymers (trace yield),^[8]^ 2) electric discharge reactions in carbon monoxide and nitrogen atmosphere (trace yield),^[9]^ and 3) the irradiation of aspartic acid and urea (~0.5% yield).^[10]^ We began a search for a milder pathway to orotate starting with an observation from a previous work demonstrating that 5-carboxymethylhydantoin could be dehydrogenated by bromination-dehydrobromination to a 5-carboxymethylidine hydantoin intermediate (II, Scheme 2), which rearranges to orotate.^[11]^ The ring expansion of this intermediate to pyrimidines, including orotate, is a known base-catalyzed transformation.^[12–15]^ Building on these works, we hypothesized that intermediate (II) could be obtained from a much simpler, and plausibly prebiotic reaction of glyoxylate with hydantoin. Indeed, when we carried out a reaction of glyoxylate with hydantoin in water at neutral pH (Scheme 2), it afforded orotate in greater than 10% yield. In a prebiotic context, hydantoin and glyoxylate are both derived from glycine, the simplest α-amino acid, by reaction with cyanate^[16]^ and formaldehyde,^[17]^ respectively. This hydantoin and glyoxylate route to orotate has been proposed previously, by Ivin et al in 1976,^[18]^ and in a subsequent 1978 US patent,^[19]^ but neither work was in the context of prebiotic chemistry. Herein, we describe and expand the scope of this reaction chemistry to demonstrate that not only is orotate produced efficiently under mild aqueous conditions, but a second metabolite, pyruvate, is also formed in significant yield from the same reactants in a single pot. Furthermore, additional chemistries, including potential nucleosidation reactions, have been identified using open-chain- and thio-hydantoin analogs.

The reaction to produce orotate began with the addition of glyoxylate to hydantoin at 60 °C and pH 8.2. After 1 h, intermediate I (Scheme 2) was produced in 75% yield, and was identifiable by ^1^H NMR as a doublet of doublets for each diastereomer (I and I*, Figure 1A). By 24 h, dehydration to the *cis*- and *trans*-5-carboxymethylidine was evident (II and II*, Figure 1B). Subsequent hydrolysis at the C4-amide generated the ring-opened intermediate III (Scheme 2). The newly liberated nitrogen (N3) then reacted with the C(7)-carboxylate to form orotate (12% yield after 7 days at 60°C, Figure S1). The proposed pathway through intermediate II is also supported by ^13^CNMR spectra of reactions starting from C4- or C5- labeled ^13^C-hydantoin, which generated orotate with ^13^C labels at carbons 7 and 6 respectively (Figures 2A, B, S2). Yields of orotate increase at higher pH and temperature; at pH values of 10 and 14, 19% and 26% orotate are produced respectively after 96 h at 80 °C (Figure 3). The reaction is tolerant to varying conditions of pH (pH 7 to 14, Figures 3, S2–S4), glyoxylate equivalents (1–10 equiv., Figures S5, S6) buffer composition (bicarbonate and phosphate, Figure S7), and hydantoin concentration (15 and 90 mM, Figure S8) demonstrating its plausibility under a range of aqueous prebiotic environments. The employed hydantoin concentrations were chosen for synthetic/analytical expediency rather than an attempt to model a hypothetical prebiotic environment. However, the hydrolytic stability of both hydantoin and glyoxylate are advantageous even at low concentrations and reaction rates, particularly under the mild reaction conditions used here.

Unexpectedly, in the reaction of hydantoin with glyoxylate, the α-keto acid pyruvate (a C3 homolog of glyoxylate) was also produced in substantial yields along with orotate (Figure 3). Pyruvate is a critical intermediate in glycolysis/gluconeogensis and the primary source of the citric acid (TCA) cycle intermediates oxaloacetate and acetyl-CoA.^[20]^ It is also an oxidant in fermentation and a carbon source for alanine biosynthesis. It has been stated that the synthesis of pyruvate may be “a critical step for the origin of life, as many extant biosynthetic pathways branch from pyruvate.”^[21]^ However, this α-keto acid has been challenging to obtain by conventional chemical pathways. Demonstrated prebiotic syntheses include: 1) the oxidation of lactate by UV irradiation over colloidal ZnS,^[22]^ or elemental sulfur,^[23]^ 2) the carbonylation of formic acid in the presence of iron sulfide and alkyl thiols at 200 MPa and 250 °C,^[21]^ and 3) the reduction of CO_2_ in the presence of iron, nickel or cobalt.^[24]^ However, the pathway herein is the first to demonstrate an efficient pyruvate synthesis (>20% yield) under mild aqueous conditions at neutral pH and moderate temperatures. The synthesis of pyruvate from glyoxylate under these conditions may also provide the building blocks and conditions necessary to sustain protometabolic networks analogous to the citric acid cycle.^[5,6,25,26]^

After 96 h at pH 8.2 and 60 °C, a 14% yield of pyruvate was observed (Figure 4A); yields above 20% were obtained at neutral pH and 80°C (Figure 3). As with orotate, the reaction chemistry is flexible to changes in pH, buffer, concentration and glyoxylate equivalents (Figures 3, S3–S8). Reaction progression through intermediates II and III to both pyruvate and orotate is supported by ^13^C-labeling studies starting from (4-^13^C) and (5-^13^C) hydantoins (Figure 4B). The pH profile of orotate and pyruvate formation (Figure 3) is consistent with a requirement for an anionic intermediate to facilitate ring closure to orotate. Although the anion of IV (Scheme 3) is likely localized on N1 rather than N3 due to increased resonance stability, the nucleophilicity of the urea group would be enhanced by either anion. Under conditions that are insufficiently basic to generate significant nitrogen anion, the reaction may alternatively progress to pyruvate through an irreversible decarboxylation of intermediate V. This decarboxylation may be accelerated by formation of an iminium intermediate,^[27]^ which has a higher probability of occurring at lower pH, thus providing an additional rationale for the pH-profile for pyruvate production seen in Figure 3. Interestingly, the presumptive intermediate VI is a ureido analog of phosphoenol pyruvate, the biosynthetic precursor to pyruvate. Hydrolysis of VI, likely again through an iminium intermediate, enables loss of urea with formation of pyruvate. Base-catalyzed hydrolyses of alkylidine hydantoins to α-keto acids have been noted in the synthesis of labeled amino acids, though no mechanism has been suggested.^[28–32]^

As an alternative to hydantoin, the reaction of 2-thiohydantoin with glyoxylate also induces ring closure to produce 2-thioorotate (Figure 2C). 2-thiohydantoin is plausibly prebiotic,^[33]^ and the reaction with glyoxylate proceeds with higher efficiency under milder conditions than hydantoin to produce 2-thioorotate in 67% yield at pH 7 after 24 h at 60°C (Figure 2C). The dependence of ring closure on the ease of nitrogen anion formation (Scheme 3) may explain the higher productivity of 2-thiohydantoin versus hydantoin in the generation of thioorotate/orotate at neutral pH; thiourea groups are typically more acidic than their urea analogs.^[34]^ 2-Thiopyrimidines, which may be generated by decarboxylation of 2-thioorotate in a reaction analogous to that shown photochemically by Ferris and Joshi for orotate,^[35]^ are observed in modern biology, including in transfer RNA, and may be of prebiotic significance.^[36]^

The rearrangement of intermediate II to orotate also suggested an alternative solution to the so called “nucleosidation problem”, wherein the coupling of fully formed nucleobases to ribose has been shown to be inefficient.^[37–38]^ If the N1-nitrogen of the hydantoin was already substituted, it should, following the pathway outlined in Scheme 2, lead to N1-substituted orotate. However, in our investigations we observed that alkylation of the N1-nitrogen of hydantoin and thiohydantoin prior to reaction with glyoxylate inhibited hydrolysis of the 5-carboxymethylidine intermediates, thus preventing formation of the orotate/thioorotate nucleoside (Figures 5A, S13–S15). A second attempt to generate the nucleoside from an open-chain analog of the substituted hydantoin (VII, Figures 5B, S16) was unsuccessful due to failure of the ring to close about the pyrimidine ring. The increased NH-acidity of the thiourea moiety^[39]^ provided the motivation to determine if thiopyrimidines might be able to complete the nucleoside formation. Thus, an open-chain thiohydantoin analog (VIII) was synthesized, which successfully cyclized to the corresponding N1-substituted thioorotate (IX, Figures 5C, D, S17, S18) in 12% yield. Although we have not demonstrated a prebiotically plausible route to VIII, it is of interest to note that IX is a building block of peptide nucleic acids (PNA), which have been proposed and investigated in the context of a pre-RNA world.^[40]^ The question of whether this would also lead to nucleoside derivatives when substituted with sugars is to be investigated in the near future.

In summary, a prebiotically plausible common pathway for the synthesis of orotate and pyruvate in 10–20% yields in a single pot has been demonstrated from two small building blocks (hydantoin and glyoxylate), both of which are plausible derivatives from a single source (glycine). These pathways are more efficient, and under milder conditions, than previously reported routes to either. The one-pot synthesis of orotate and pyruvate is significant when juxtaposed with how these are formed in extant biology. Orotate is biosynthesized from aspartic acid and converted to orotidine by reaction of the nucleobase orotate with 5′-phosphoribosyl-1′-pyrophosphate (PRPP, Scheme 1). Subsequent decarboxylation of the orotidine intermediate yields uridine, followed by amination to cytidine. In the absence of UV photolysis,^[35]^ the uncatalyzed rate of this decarboxylation has a half-time of nearly 80 million years.^[41]^ A highly efficient enzyme, orotidylate decarboxylase, accelerates the reaction by a factor of 10^17^ in order to maintain a sufficient physiological supply of pyrimidines (Scheme 1).^[41]^ The inclusion of this difficult metabolic step may indicate that contemporary pyrimidine biosynthesis recapitulates an evolutionary progression of orotate from a functional nucleobase into its current role as a biosynthetic intermediate to modern nucleosides, once capable enzymes evolved for its decarboxylation although other rationales have been proposed.^[42]^ Alternatively, if a UV-photolytic environment enabling decarboxylation was compatible with an emerging protometabolism, orotate may have provided a source of pyrimidines, as it does in modern biology.^[43]^ The plausibility of either proposal is enhanced by the identification of prebiotic sources of orotate,^[43]^ and the results reported here, with yields up to 20%, provide support for a robust prebiotic role for orotate. Additionally, if pyruvate served as a source of sugars in an early biotic world, as it does in modern biochemistry, the production of significant amounts of pyruvate along with orotate in a single pot may have provided the building blocks towards nucleosidation with ribose or related sugars, as well as supported additional compatible pyruvate-dependent metabolic pathways. Thus, the identification of conditions that enable syntheses of orotate and pyruvate in one pot may increase the plausibility of co-origination and co-evolution of protometabolic pathways, nucleic acid synthesis, and systems prebiotic chemistry. Further explorations into the reaction chemistry are seeking ways to connect, in a single pot, protometabolic reaction pathways to even smaller C1 building blocks including formaldehyde, cyanate and cyanide.

## Supplementary Material

Supplementary Material

## Figures and Tables

**Figure 1.**
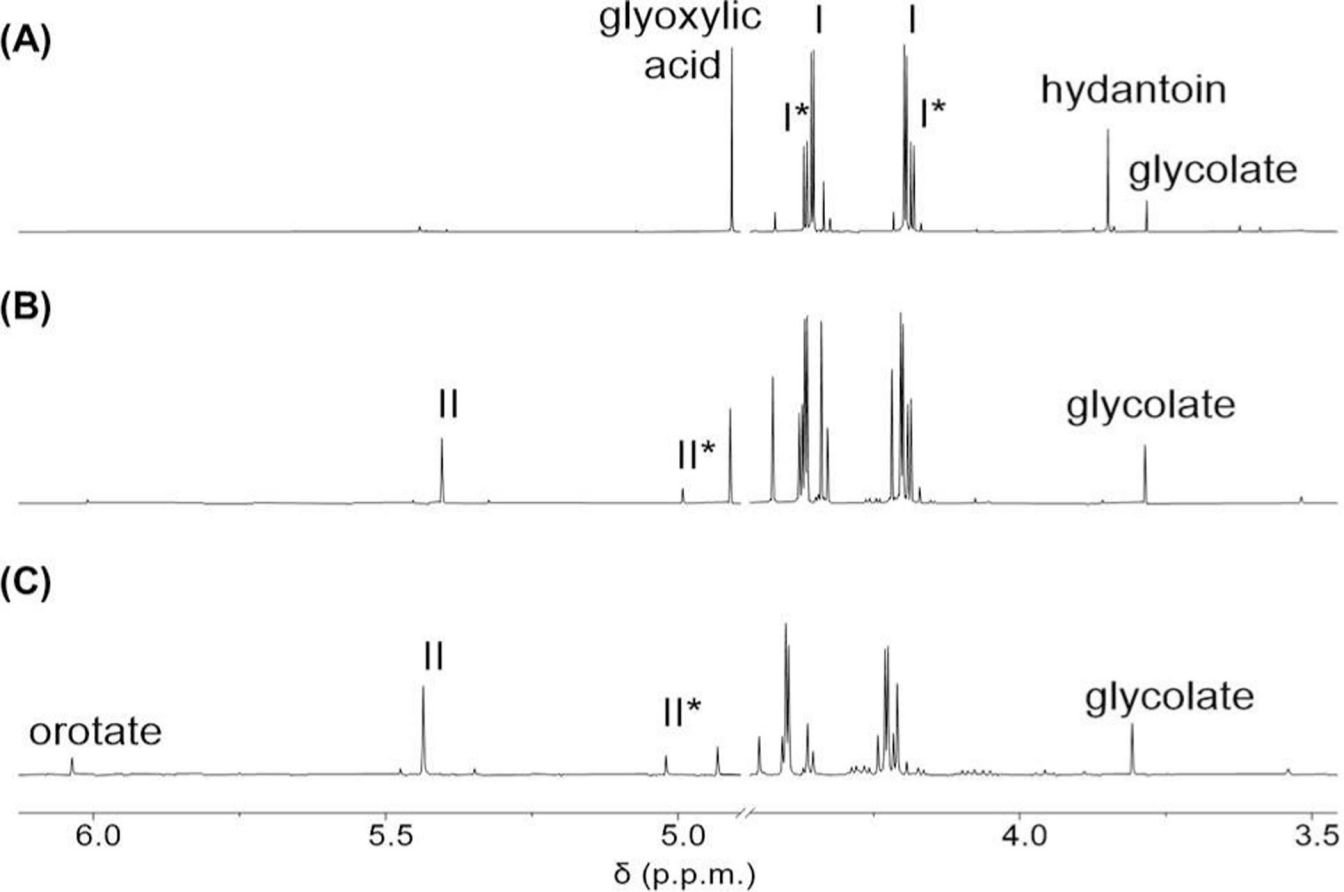
The proposed pathway from hydantoin to orotate. ^1^H NMR (in D_2_O) of a reaction aliquot from 90 mM hydantoin with 1.5 eq. of glyoxylic acid in pH 8.2 1.0 M NaHCO_3_ buffer stirred at 60 °C for 1 hour (A), for 24 hours (B), and for 96 hours (C).

**Figure 2.**
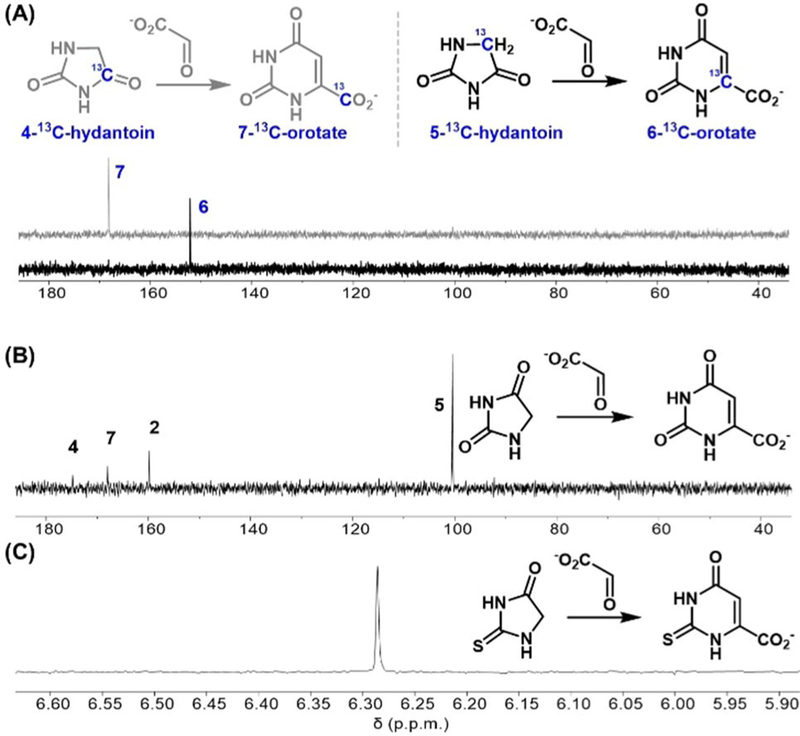
^13^C NMR (in D_2_O) showing A) (7-^13^C)orotate and (6-^13^C)orotate produced from C4- and C5-^13^C-hydantoins, respectively, B) in comparison to a non isotopically-labeled orotate. C) ^1^H NMR (in D_2_O) of a reaction aliquot from 50 mM 2-thiohydantoin with 1.5 eq. of glyoxylic acid in a pH 7 0.5 M NaH_2_PO_4_ buffer stirred at 60 °C to produce 2-thioorotate.

**Figure 3.**
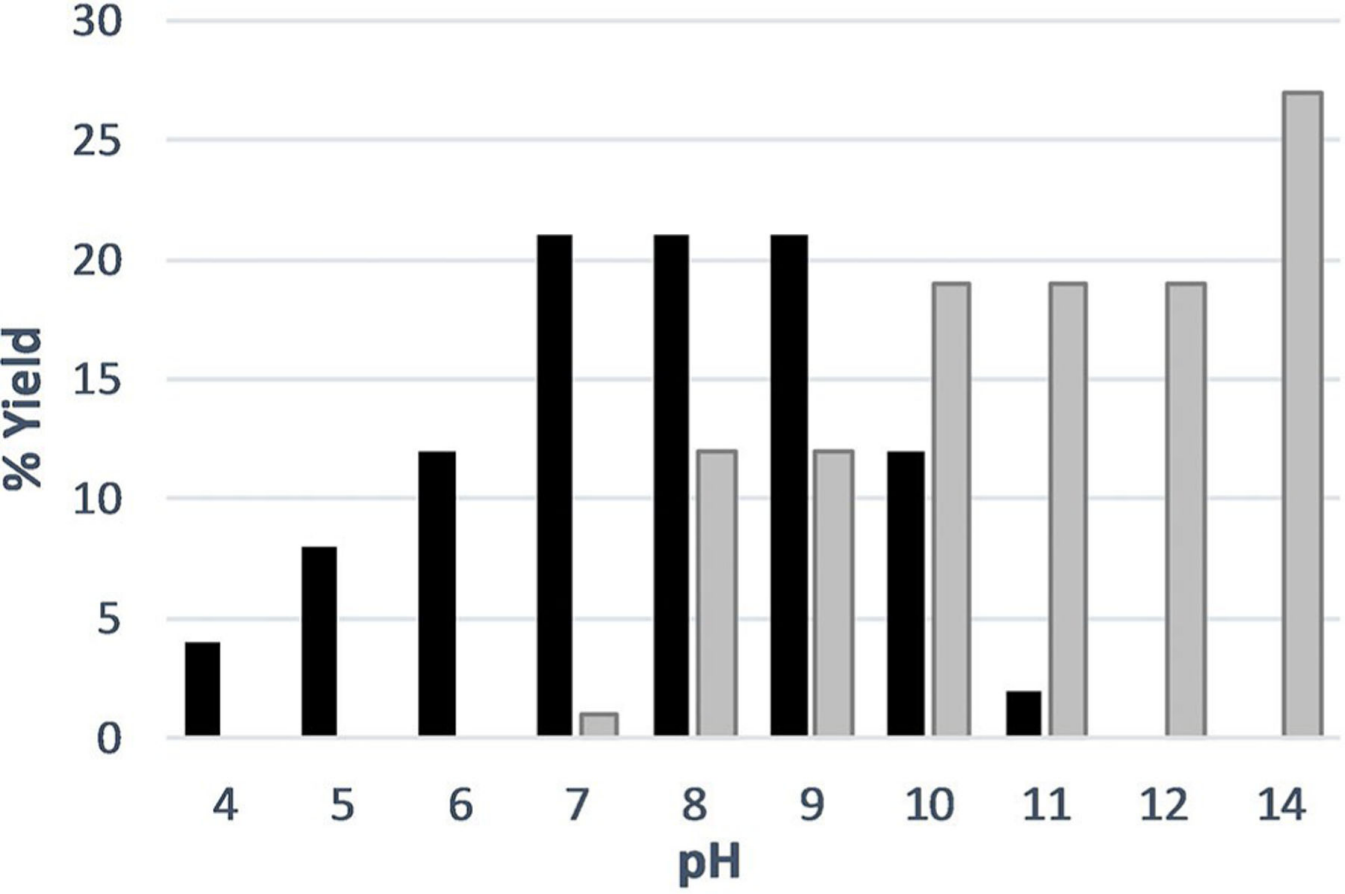
pH dependence of pyruvate (black) and orotate (shaded) production from 90 mM hydantoin with 1.5 eq. of glyoxylic acid, heated at 80 °C for 96 h in phosphate buffer (pH 4–7, 11–12), bicarbonate buffer (pH 8–10), or 2.0 M KOH (pH 14).

**Figure 4.**
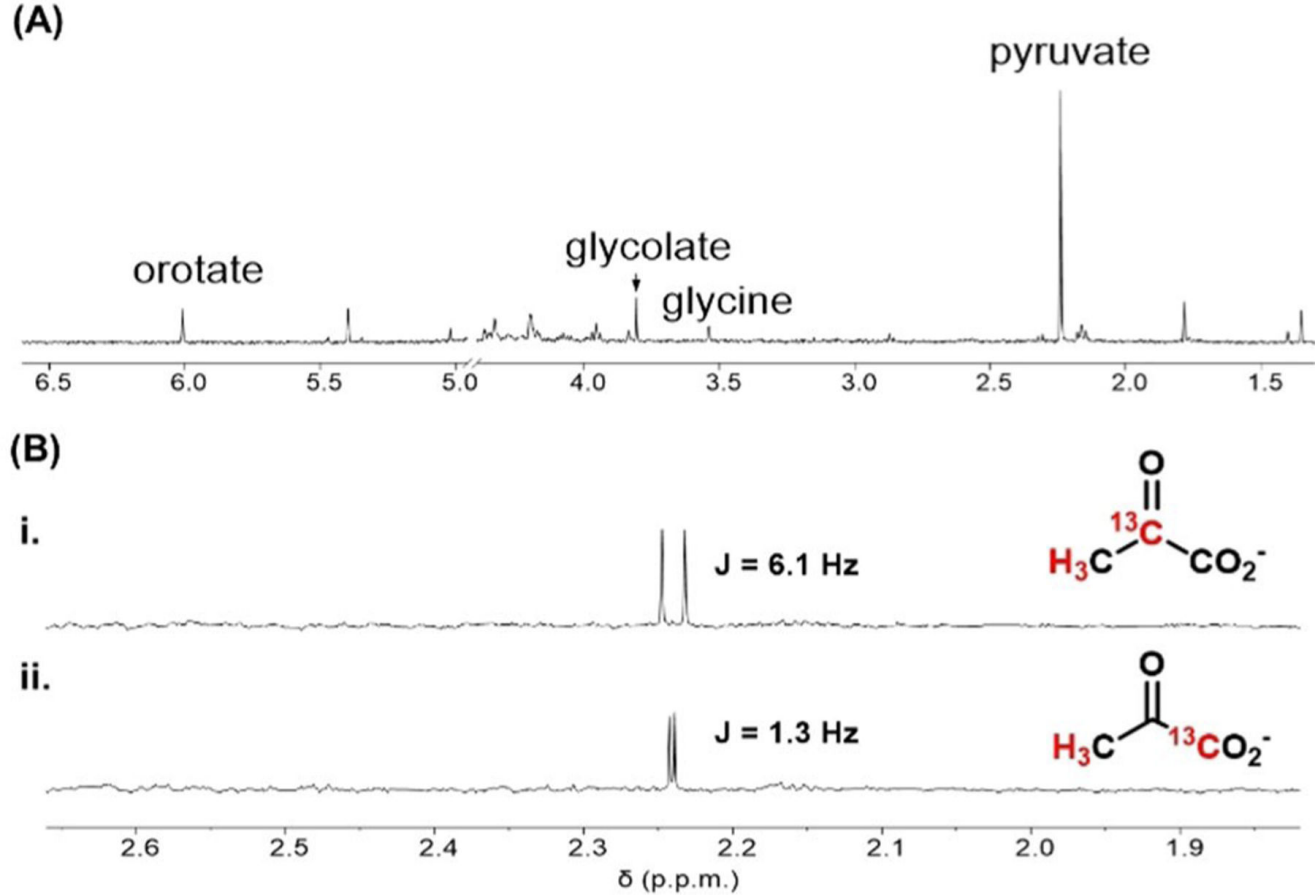
A) Synthesis of pyruvate in 1.0 M NaHCO_3_, pH 8.2, 60 °C for 96 h. B) Pyruvate produced from i) (5-^13^C)hydantoin and ii) (4-^13^C)hydantoin in 1.0 M NaHCO_3_ pH 8.2, with 1.5 eq. of glyoxylic acid heated at 60 °C for 72 h. C–H couplings are consistent with expected values for i. two-bond and ii. three-bond proton-carbon couplings.

**Figure 5.**
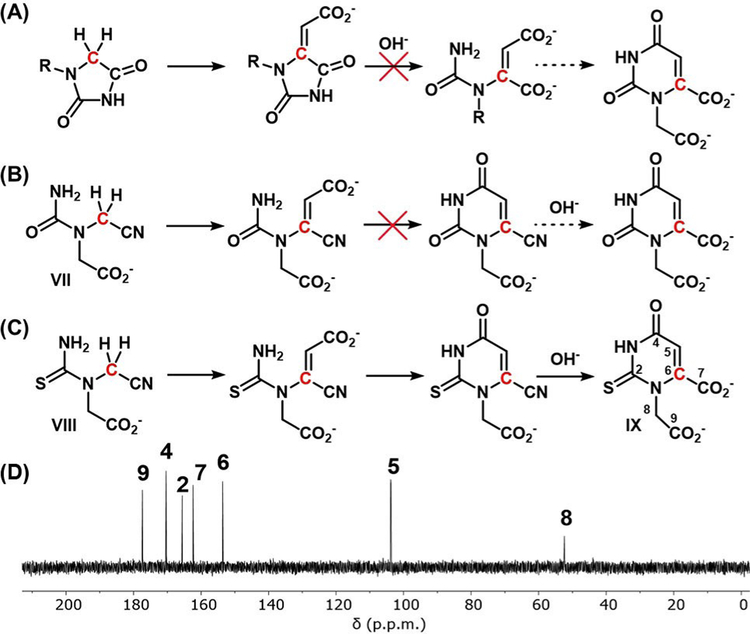
Nucleosidation of hydantoin analogues. Attempts to synthesize orotate nucleosides with 1.5 equiv. of glyoxylate in 1 M NaHCO_3_ at 80 °C were unsuccessful starting from: A) N1-alkylated hydantoins, and B) open-chain hydantoin analogues (VII). C) However, an open-chain thiohydantoin analogue (VIII) was successfully transformed into a carboxymethylthiorotate nucleoside (IX), as shown by its ^13^CNMR (D).

**Scheme 1.**
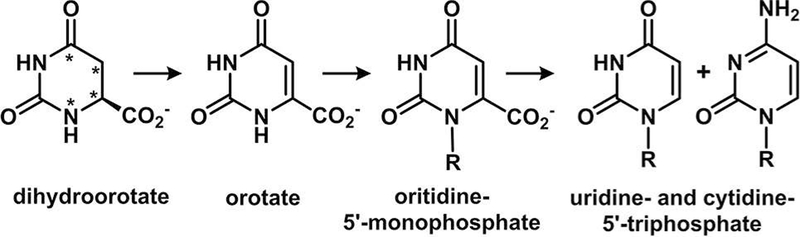
The biosynthesis of pyrimidine nucleotides proceeds through an orotate intermediate that is subsequently ribosylated and decarboxylated to produce the canonical uridine and cytidine nucleotides. The original aspartate skeleton within dihydroorotate is starred.

**Scheme 2.**
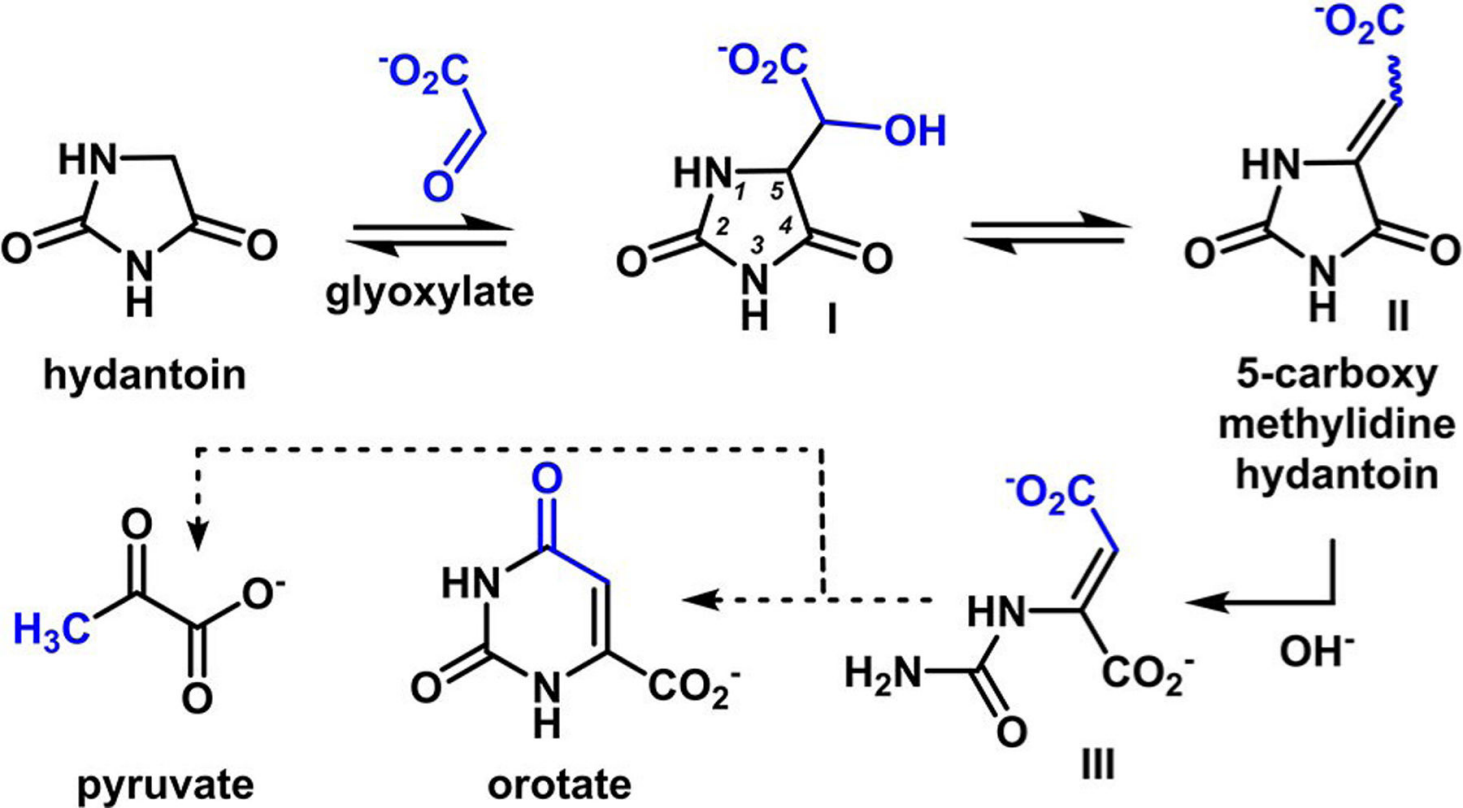
The reaction pathway from hydantoin and glyoxylate proceeds through the hydrolysis of 5-carboxymethylidinehydantoin (II) to intermediate III. Attack of the liberated N3 on the carboxylate (C7) produces orotate. Tautomerization and hydrolysis of III yields pyruvate. The numbering system maintains consistency from hydantoin, and the blue color tracks the glyoxylate skeleton.

**Scheme 3.**
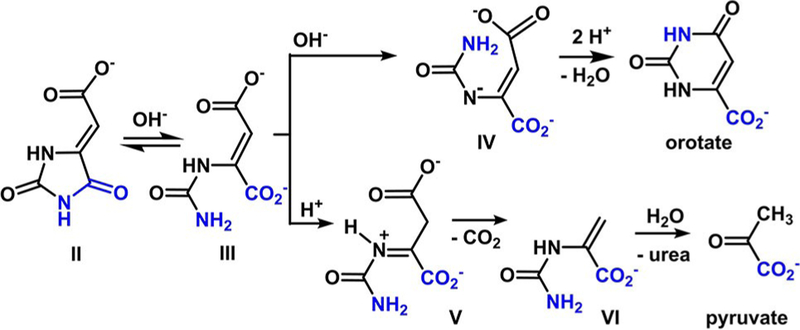
Bifurcation of the mechanistic pathway to orotate and pyruvate from 5-carboxymethylidinehydantoin (II).
