# Supplementary material for: A Plausible Prebiotic One-Pot Synthesis of Orotate and Pyruvate Suggestive of Common Protometabolic Pathways
Source: Angew Chem Int Ed Engl. Author manuscript; Available in PMC 2022 Mar 7. (PMC8885966; doi:10.1002/anie.202112572)
Supplement: Supplementary Material [file NIHMS1771883-supplement-Supplementary_Material.pdf]

Angewandte Chemie
Eine Zeitschrift der Gesellschaft Deutscher Chemiker

Supporting Information

A Plausible Prebiotic One-Pot Synthesis of Orotate and Pyruvate Suggestive of Common Protometabolic Pathways

A. P. Clay, R. E. Cooke, R. Kumar, M. Yadav, R. Krishnamurthy, G. Springsteen**

Supporting Information

Author Contributions

R.K. and G.S. conceived the project. A.P.C., R.E.C, M.Y., G.S., and R.K. proposed, designed, and carried out experiments. All authors interpreted the data and discussed the experimental results. R.K., M.Y. and G.S. supervised the research and wrote the paper with comments and feedback from A.P.C. and R.E.C.

Table of Contents

Experimental Procedures 3

Synthetic Methods: 3

Figure S1. Synthesis of (7-^{13}C)orotate....................5

Figure S2. pH dependence of orotate synthesis....................6

Figure S3. pH dependence for orotate and pyruvate yields in acidic and neutral conditions. 7

Figure S4. pH dependence for orotate and pyruvate yields in basic conditions....................8

Figure S5. The effect of glyoxylate equivalents on orotate and pyruvate yield at pH 13.5.........9

Figure S6. The effect of glyoxylate equivalents on orotate and pyruvate yield at pH 7.0.........10

Figure S7. Comparison of buffer dependence on orotate, uracil and pyruvate yields................11

Figure S8. Effect of hydantoin concentrations on the production of pyruvate and orotate.........12

Figure S9. Coupling constants for 7-^{13}C)orotate and (6-^{13}C)orotate. 13

Figure S10. (4-^{13}C)hydantoin synthesis....................14

Figure S11. (5-^{13}C)hydantoin synthesis....................15

Figure S12. Aldol addition of hydantoin and glyoxylate to intermediate **I** occurs at a range of pH values....................16

Figure S13. Orotate nucleoside synthesis halts at 5-carboxymethylidine-hydantoins.............17

Figure S14. *E* and *Z* 5-carboxymethylidine-hydantoins. 18

Figure S15. Attempted synthesis of 1-methyl-2-thioorotate nucleoside derivative..................19

Figure S16. Attempted synthesis of 1-carboxymethyl orotate nucleoside derivative. 20

Figure S17. Synthesis of 1-carboxymethyl-2-thioorotate. 21

Figure S18. HPLC standard curve generated for 1-carboxymethyl-2-thioorotate.. 22

Experimental Procedures

The following reagents were purchased and used as received: 50 wt% in H_2O glyoxylic acid solution (Sigma-Aldrich 260150), hydantoin (98%, Sigma-Aldrich 156361), orotic acid monohydrate (97%, Sigma-Aldrich O84020), sodium pyruvate (99%, Sigma Aldrich P2256), (2-^{13}C)glycine (99%, Sigma-Aldrich 279439), (1-^{13}C)glycine (99%, Sigma-Aldrich 279420), and 2-thiohydantoin (99%, Sigma-Aldrich T30406).

^{1}H NMR and ^{13}C NMR spectra were measured using a Varian 400-MR, INOVA 500 and AV-600 (equipped with cryoprobe) spectrometer. Reaction progress was monitored using ^{1}H NMR unless otherwise noted. Chemical shifts are presented in ppm. ^{1}H NMR spectra were referenced using the residual solvent peak (δ(D_2O) 4.79). Product yields were measured by ^{1}H NMR integration compared with an internal standard (t-butanol) in D_2O NMR solvent. Yields are calculated as the percent of the theoretical yield from limiting reactant (non-isolated). Pyruvate yield was calculated by combining the ^{1}H NMR integration of pyruvate and pyruvate hydrate as compared with the internal standard.

pH values were determined using a calibrated Mettler Toledo InLab Expert Pro probe. Analytical reverse-phase HPLC was performed on two different instruments. First is Waters Alliance instrument equipped with an e2695 XC Separations Module w/CHC. UV spectroscopic data was measured using a 2998 Photodiode Array Detector. Second is a Thermofisher Ultimate 3000 (mentioned wherever used) equipped with a Photodiode Array Detector. HPLC Mass spectroscopic data was obtained on an AQUITY QDa Mass Detector. Separation was achieved using a Synergi 4 µm Polar-RP 80 Å, LC column (Phenomenex) at a flow rate of 0.750 mL/min using an isocratic mobile phase of 95% 0.1% v/v formic acid buffer pH 2.8 and 5% methanol. However, with the Thermofisher HPLC, HPLC experiments were performed with the NUCLEOSIL 100-5 C18 column (5 µm, 4.6 x 250 mm) by using gradient mobile phase i.e., 0 – 50% B over 10 min (Buffer A = 0.01 M TEAA, pH 7; Buffer B = 25% H_2O in CH_3CN; flow rate = 1.0 mL/min). HRMS spectra for selected compounds were recorded on a mass spectrometer at the Scripps Mass Spectrometry Center.

Synthetic Methods:

General procedure for orotate production under various pH and buffer conditions. 90 mg of hydantoin (90 mM) was dissolved in 9.9 mL of appropriate buffer in a 20-mL scintillation vial with a stir bar. 149 µL (1.5 eq) of glyoxylic acid (50 wt% in H_2O) was added using a micropipette. The reaction pH was adjusted accordingly using 4.0 M aq. NaOH or 4.0 M HCl. The reaction vial was then placed on a magnetic stir plate fitted with a vial reaction block heater and stirred for 96 h under an N_2 atmosphere at either 60 °C to best observe reaction intermediates, or 80 °C to maximize product yields.

Orotate. 90 mg of hydantoin (90 mM) was dissolved in 9.9 mL of 1.0 M $NaHCO_3$ in a 20-mL scintillation vial with a small stir bar. 149 µL (1.5 eq) of glyoxylic acid (50 wt% in H_2O) was added using a micropipette. The reaction pH was adjusted to 8.2 using 4.0 M aq. NaOH. The reaction vial was then placed on a magnetic stir plate fitted with a vial reaction block heater and stirred for 96 h at 80 °C (or 7 days at 60 °C) under an N_2 atmosphere to provide orotate (12% yield). ^{1}H NMR, 400 MHz, D_2O – δ 6.02 ppm (s, 1H).

Pyruvate. 90 mg of hydantoin (90 mM) was dissolved in 9.9 mL of 1.0 M $NaHCO_3$ in a 20-mL scintillation vial with a small stir bar. 149 µL (1.5 eq) of glyoxylic acid (50 wt% in H_2O) was added using a micropipette. The reaction pH was adjusted to 8.2 using 4.0 M aq. NaOH. The reaction vial was then placed on a magnetic stir plate fitted with a vial reaction block heater and stirred for 96 h at 80 °C under an N_2 atmosphere to provide pyruvate (21% yield). Alternatively, stirring for 96 h at 60 °C yields 14% pyruvate. ^{1}H NMR, 400 MHz, D_2O – *pyruvate:* δ 2.24 ppm (s, 3H). *pyruvate hydrate:* δ 1.47 ppm (s, 3H).

(4-^{13}C)hydantoin. 1.00 g (1-^{13}C)glycine (822 mM) was dissolved in 16 mL DI water in a 100 mL round bottom flask. 1.40 g of potassium cyanate (1.3 eq) was added and the reaction was heated at reflux for 3 h. After the (1-^{13}C)glycine had been consumed, 4.8 mL of 12.1 M HCl was added to the reaction flask stirring over an ice-water bath over 5 minutes. The reaction was then heated at reflux for 3.5 h. The reaction flask was chilled at 4 °C for 12 hours. Crystals were then collected using vacuum filtration and washed with a minimal amount of cold DI water. (4-^{13}C)hydantoin was recovered with 57% yield. ^{1}H NMR, 400 MHz, D_2O – *(1-^{13}C)hydantoic acid:* δ 3.52 ppm (d, 2H). *(4-^{13}C)hydantoin:* δ 3.93 ppm (d, 2H).

(5-^{13}C)hydantoin. 1.00 g (2-^{13}C)glycine (822 mM) was dissolved in 16 mL DI water in a 100 mL round bottom flask. 1.40 g of potassium cyanate (1.3 eq) was added to the flask, and the reaction was heated at reflux for 3 h. After all the (2-^{13}C)glycine had been consumed, 4.8 mL of 12.1 M HCl was added to the reaction flask stirring over an ice-water bath over 5 minutes. The reaction was then heated at reflux for 3.5 h. The reaction flask was chilled at 4 °C for 12 hours. Crystals were then collected using vacuum filtration and washed with a minimal amount of cold DI water. (5-^{13}C)hydantoin was recovered with 57% yield. ^{1}H NMR, 400 MHz, D_2O – *(2-^{13}C)hydantoic acid:* δ 3.52 ppm (d, 2H). *(5-^{13}C)hydantoin:* δ 3.93 ppm (d, 2H).

(6-^{13}C)orotate. 91 mg of (5-^{13}C)hydantoin (90 mM) was dissolved in 9.9 mL of 1.0 M $NaHCO_3$ in a 20-mL scintillation vial with a small stirbar. 149 µL (1.5 eq) of glyoxylic acid (50 wt% in H_2O) was added using a micropipette. The reaction pH was adjusted to 8.0 using 4.0 M aq. NaOH. The reaction vial was then placed on a magnetic stir plate fitted with a vial reaction block heater and stirred for 8 days at 60 °C to give (6-^{13}C)orotate with 15% yield. ^{1}H NMR, 400 MHz, D_2O – δ 6.02 ppm (d, 1 H).

(7-^{13}C)orotate. 91 mg of (4-^{13}C)hydantoin (90 mM) was dissolved in 9.9 mL of 1.0 M $NaHCO_3$ in a 20-mL scintillation vial with a small stirbar. 149 µL (1.5 eq) of glyoxylic acid (50 wt% in H_2O) was added using a micropipette. The reaction pH was adjusted to 8.0 using 4.0 M aq. NaOH. The reaction vial was then placed on a magnetic stir plate fitted with a vial reaction block heater and stirred for 8 days at 60 °C to give (7-^{13}C)orotate with 15% yield. ^{1}H NMR, 400 MHz, D_2O – δ 6.02 ppm (d, 1 H).

(1-^{13}C)pyruvate. 91 mg of (4-^{13}C)hydantoin (90 mM) was dissolved in 9.9 mL of 1.0 M $NaHCO_3$ in a 20-mL scintillation vial with a small stirbar. 149 µL (1.5 eq) of glyoxylic acid (50 wt% in H_2O) was added using a micropipette. The reaction pH was adjusted to 8.0 using 4.0 M aq. NaOH. The reaction vial was then placed on a magnetic stir plate fitted with a vial reaction block heater and stirred for 72 hours at 60 °C to give *(1-^{13}C)pyruvate.* ^{1}H NMR, 400 MHz, D_2O – *(1-^{13}C)pyruvate:* δ 2.24 ppm (d, 3H).

(2-^{13}C)pyruvate. 91 mg of (5-^{13}C)hydantoin (90 mM) was dissolved in 9.9 mL of 1.0 M $NaHCO_3$ in a 20-mL scintillation vial with a small stirbar. 149 µL (1.5 eq) of glyoxylic acid (50 wt% in H_2O) was added using a micropipette. The reaction pH was adjusted to 8.0 using 4.0 M aq. NaOH. The reaction vial was then placed on a magnetic stir plate fitted with a vial reaction block heater and stirred for 72 hours at 60 °C to give *(2-^{13}C)pyruvate.* ^{1}H NMR, 400 MHz, D_2O – *(2-^{13}C)pyruvate:* δ 2.24 ppm (d, 3H).

2-thioorotate. 58 mg of 2-thiohydantoin (50 mM) was dissolved in 9.9 mL of 0.5 M aq. pH 7.0 sodium phosphate buffer in a 20-mL scintillation vial with a small stir bar. 83 µL (1.5 eq) of glyoxylic acid (50 wt% in H_2O) was added using a micropipette. The reaction pH was adjusted to 7.0 using 4.0 M aq. NaOH. The reaction vial was then placed on a magnetic stir plate fitted with a vial reaction block heater and stirred for 24 h at 60 °C to give 2-thioorotate with 67% yield. ^{1}H NMR, 400 MHz, D_2O – δ 6.29 ppm (s, 1H).

Synthesis of 1-carboxymethyl-2-thioorotate:

1-cyanomethyl-1-(carboxymethyl)thiourea (VIII). 143 mg (500 mM) iminodiacetonitrile was dissolved in 3 mL 2.0 M HCl. 171 mg (1.5 eq.) ammonium thiocyanate was added, and the reaction was stirred at 80 °C for 3 h to give the desired product. This reaction mixture was used directly for the next step as described below. ^{1}H NMR, 400 MHz, D_2O – δ 4.25 ppm (s, 2H), 4.45 ppm (s, 2H).

1-carboxymethyl-2-thioorotate (IX). The crude **VIII** was neutralized with solid $NaHCO_3$. The reaction was then diluted with 47 mL of 1.0 M $NaHCO_3$ to a total volume of 50 mL. 414 µL of glyoxylic acid (50 wt% in H_2O) was added and the reaction was heated at 80 °C for 3 days. The reaction mixture was then cooled, and the solvent was removed using rotary evaporation. 50 mL of acetone was added, and the crude mixture was stirred at RT for 15 minutes. The acetone was then decanted, and the remaining solid was acidified to pH 1 using 4 M HCl (~25 mL) which provided a clear solution of the crude material. Concentration of this clear solution under vacuum (~40 °C) afforded the crude material along with lot of NaCl. Then, 50 mL of ethanol was added to the crude, stirred for 15 minutes, and filtered. Filtrate was then concentrated using rotary evaporation, affording 450 mg of an orange solid which was purified using ion-exchange chromatography (as described below) to give pure 1-carboxymethyl-2-thioorotate (12% absolute yield, determined by HPLC analysis described below). ^{1}H NMR, 400 MHz, D_2O – δ 6.16 ppm (s, 1H). ^{13}C NMR, 400 MHz, D_2O – δ 63.09, 105.51, 152.37, 162.46, 165.53, 171.01, 176.96 ppm. HRMS (ESI/Q-TOF) m/z calculated for $C_7H_6N_2O_5S$ $[M-H]^-$ 228.9925, found 228.9927.

Ion exchange Chromatography of 1-carboxymethyl-2-thioorotate: The crude product (concentrated ethanol fraction) was dissolved in minimum amount of water (1-2 mL) and loaded on the Sephadex ion-exchange column (DEAE-A-25-sephadex, 40-120 mesh, HCO_3^- form, 1.5 x 7.0 cm). After that, the column was first eluted with 100 mL of distilled water (to remove all the uncharged species), followed by elution with 0.04M – 1.0M $NaHCO_3$ in 0.04 M increments of 20 mL. The fractions containing the pure product (monitored by HPLC) were combined, neutralized with Amberlite IR 120 H^+ (in case of $NaHCO_3$ buffer) and concentrated to dryness at 35 °C under vacuo to afford the pure compound. The required compound was eluted in ~ 0.3 M bicarbonate buffer.

Preparation of the Sephadex ion-exchange resin (bicarbonate form): Ion exchange resin DEAE–Sephadex® A-25 chloride form (*Sigma Aldrich, A25120*), 20 g was dissolved in 300 mL of saturated $NaHCO_3$ solution in a 500 mL conical flask and shook on a shaker for overnight (12-24 hrs). After that the resin was filtered on a sintered funnel and was washed with distilled water several times (6-7) until pH of the filtrate water is ≤7.0. Now the Sephadex resin is in bicarbonate from, which could be stored in distilled water and can be used for the purification of the required compound. **[***Note:* Please do not stir the Sephadex resin, stirring may damage the resin. During filtration process (after treatment with sat. $NaHCO_3$ solution), try not to dry the resin completely. After filtration, the resin should be stored in distilled water (it will remain good over a period of 6 months)].

Determination of yield of 1-carboxymethyl-2-thioorotate: Purified fraction of 1-carboxymethyl-2-thioorotate with known concentration (determined by ^{1}H NMR analysis using uridine as an internal standard) was serial diluted to generate a standard HPLC curve which was further utilized to determine the absolute yield of the reaction (Figure S18).

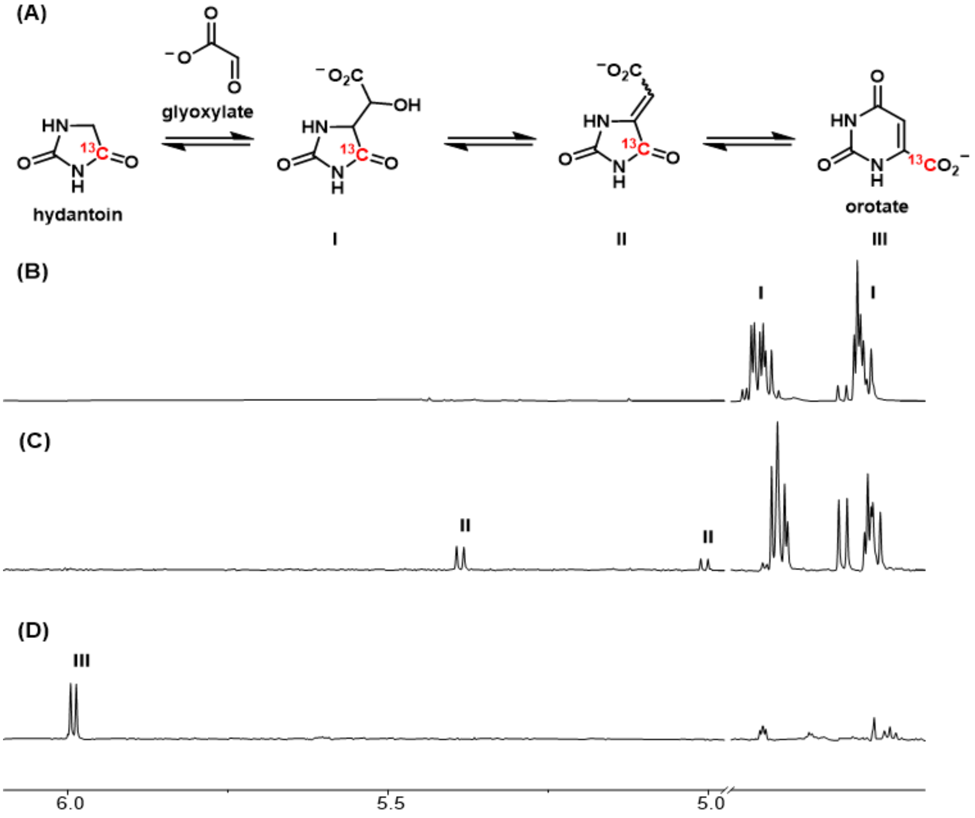

Figure S1. (A) Synthesis of (7-^{13}C)orotate. **(B)** ^{1}H NMR (in D_2O) of a reaction aliquot from 90 mM (4-^{13}C)hydantoin in 1.0 M $NaHCO_3$, pH 8.2, with 1.5 eq. of glyoxylic acid, stirred at 60 °C for 1 hour, **(C)** for 24 hours, and **(D)** for 8 days.

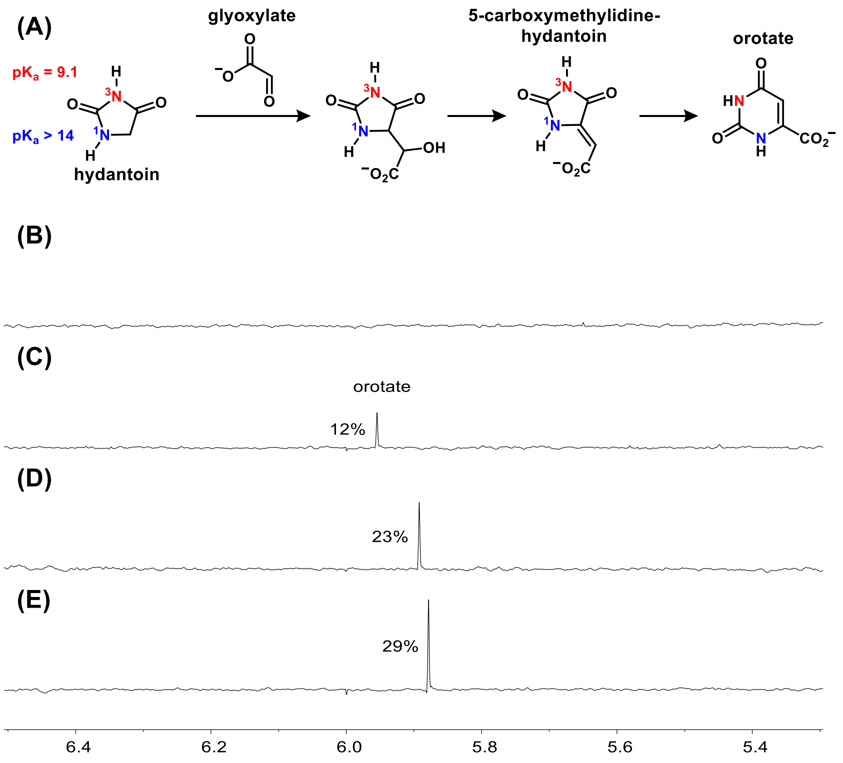

Figure S2. pH dependence of orotate synthesis. **(A)** Orotate generation from hydantoin and glyoxylate. **(B)** ^{1}H NMR (in D_2O) of a reaction aliquot from 90 mM hydantoin with 1.5 eq. of glyoxylic acid, stirred at 60 °C for 7 days in 1.0 M aq. phosphate buffer, pH 5.5, (**C**) in 1.0 M aq. $NaHCO_3$, pH 8.2, **(D)** in aq. sat. Na_2CO_3, pH 11, and **(E)** in 1.0 M aq. KOH, pH 13.5.

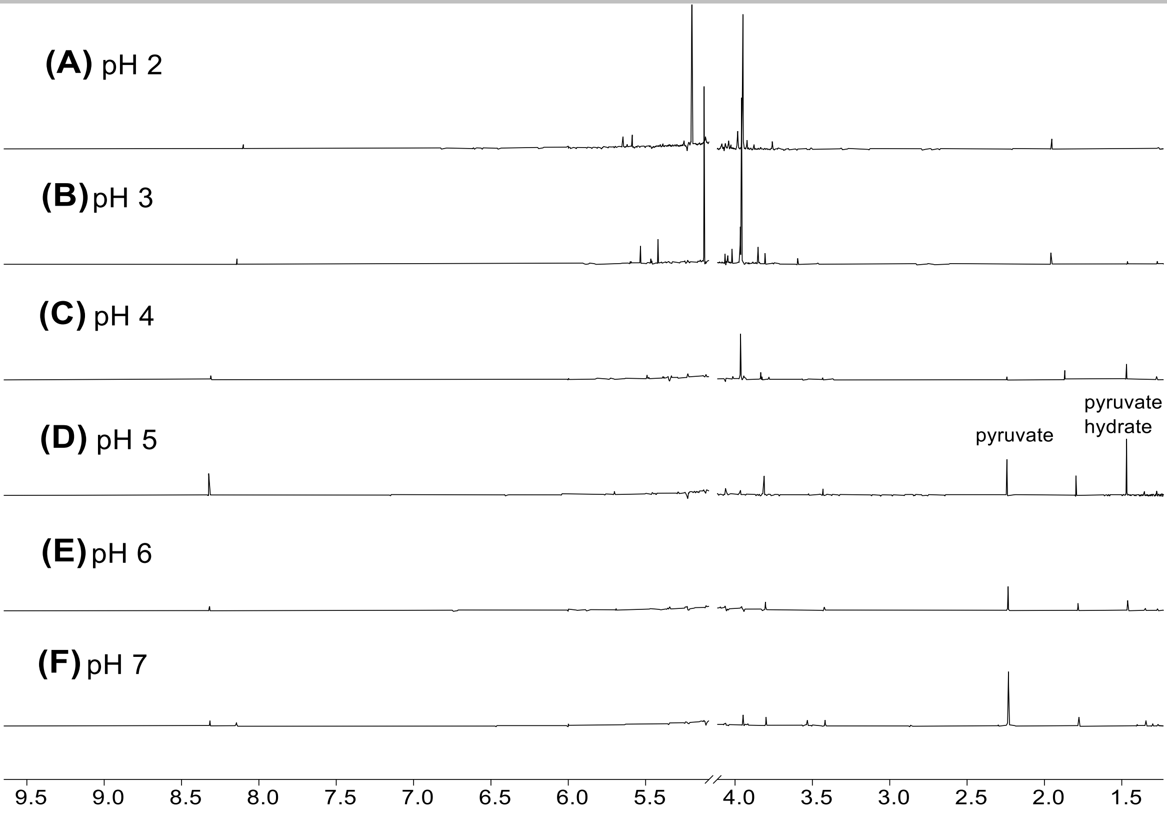

Figure S3. pH dependence for orotate and pyruvate yields in acidic and neutral conditions. ^{1}H NMR in D_2O of reaction aliquot from 90 mM hydantoin with 1.5 eq. of glyoxylic acid, stirred at 80 °C for 96 hours. **(A)** 0.5 M aq. phosphate buffer, **(B)** 0.5 M aq. phosphate buffer, **(C)** 1.0 M aq. phosphate buffer, **(D)** 1.0 M aq. phosphate buffer, **(E)** 0.5 M aq. phosphate buffer, and **(F)** 0.5 M aq. phosphate buffer.

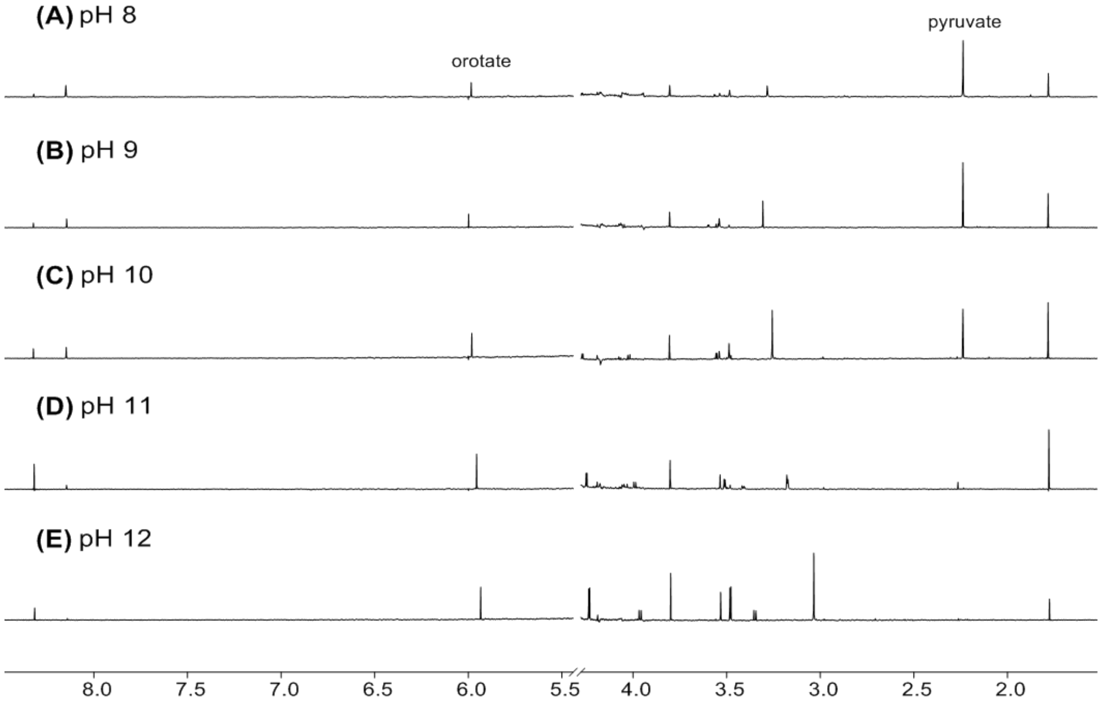

Figure S4. pH dependence for orotate and pyruvate yields in basic conditions. ^{1}H NMR in D_2O of reaction aliquot from 90 mM hydantoin with 1.5 eq. of glyoxylic acid, stirred at 80 °C for 96 hours. **(A)** 1.0 M aq. bicarbonate buffer, **(B)** 1.0 M aq. carbonate buffer, **(C)** 1.0 M aq. carbonate buffer, **(D)** 0.5 M aq. phosphate buffer, and **(E)** 0.5 M aq. phosphate buffer.

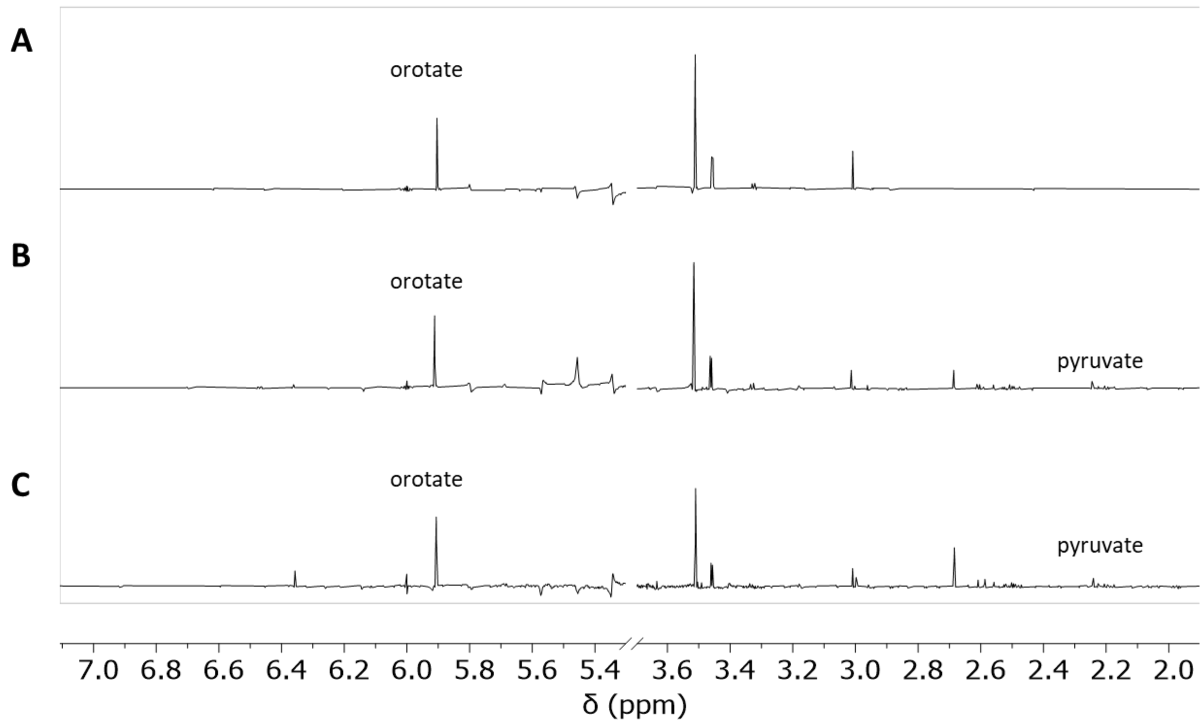

Figure S5. The effect of glyoxylate equivalents on orotate and pyruvate yield at pH 13.5. ^{1}H NMR spectra in D_2O of a reaction aliquot from 90 mM hydantoin in 1.0 M KOH, pH 13.5, stirred at 60 °C for 7 days with (**A**) 1 eq. glyoxylate, **(B)** 3 eq. glyoxylate, **(C)** 10 eq. glyoxylate, indicating all three reactions produced a 20% yield of orotate. Trace amounts of pyruvate are observed at 3 and 10 equivalents of glyoxylate.

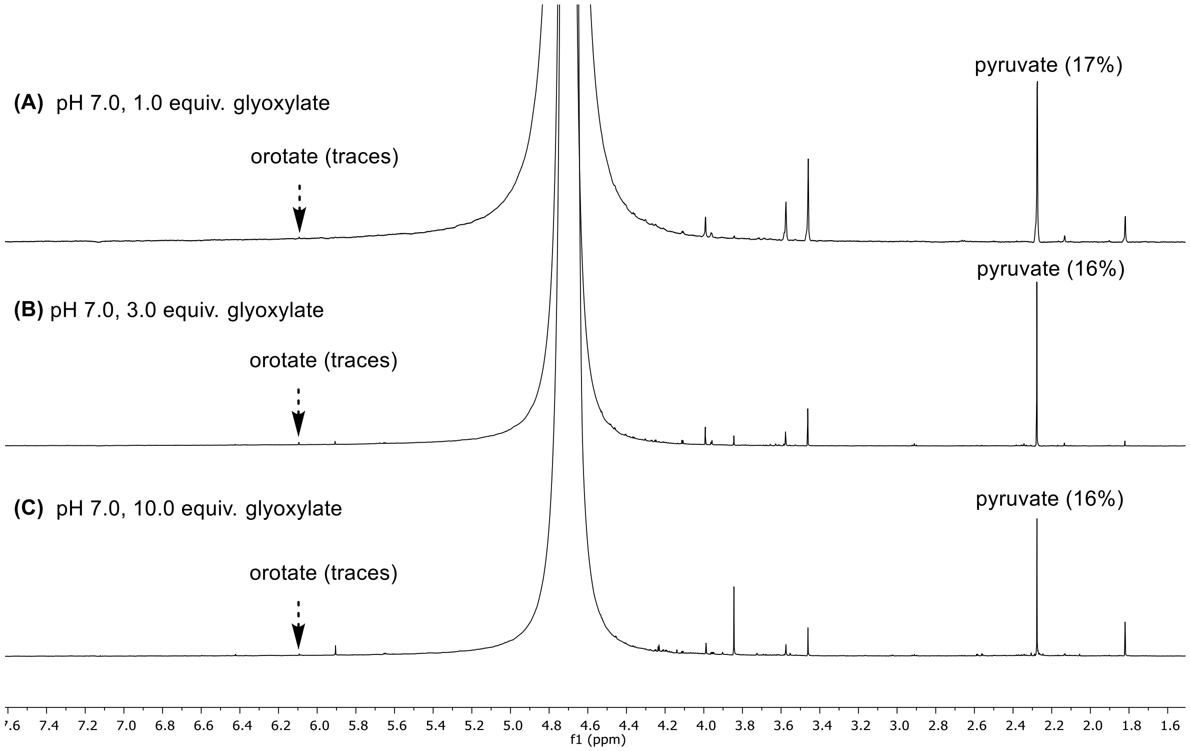

Figure S6. The effect of glyoxylate equivalents on orotate and pyruvate yield at pH 7.0, 0.5 M phosphate buffer stirred at 80 °C for 3 days. ^{1}H NMR (in D_2O) of a reaction aliquot with **(A)** 1 eq. glyoxylate, **(B)** with 3 eq. glyoxylate, **(C)** with 10 eq. glyoxylate, yielded 16-17% pyruvate and traces of orotate. pH values corrected with aq. NaOH after glyoxylate addition.

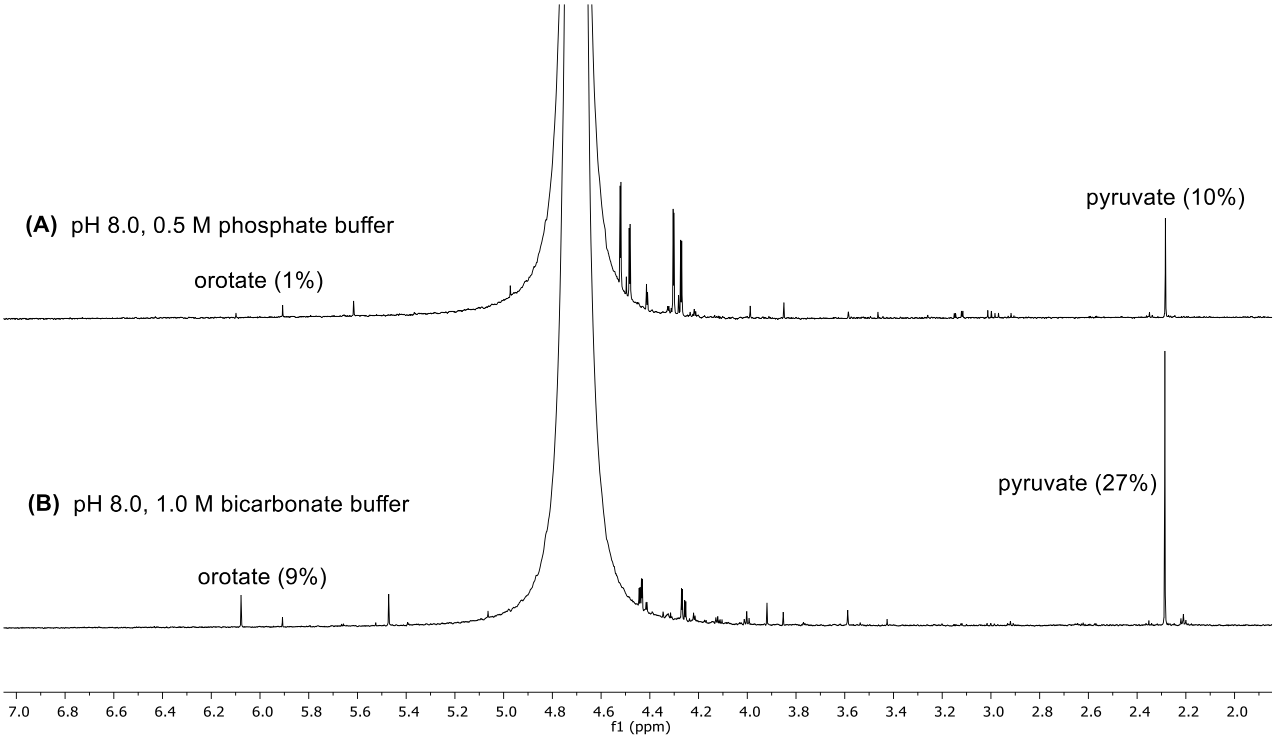

Figure S7. Comparison of buffer dependence on orotate, uracil and pyruvate yields. ^{1}H NMR in D_2O of reaction aliquot from 90 mM hydantoin with 1.5 eq. of glyoxylic acid, stirred at 60 °C for 7 days. **(A)** 0.5 M aq. phosphate buffer at pH 8 (final pH = 7.8), and **(B)** 1.0 M aq. bicarbonate buffer at pH 8 (final pH = 8.2).

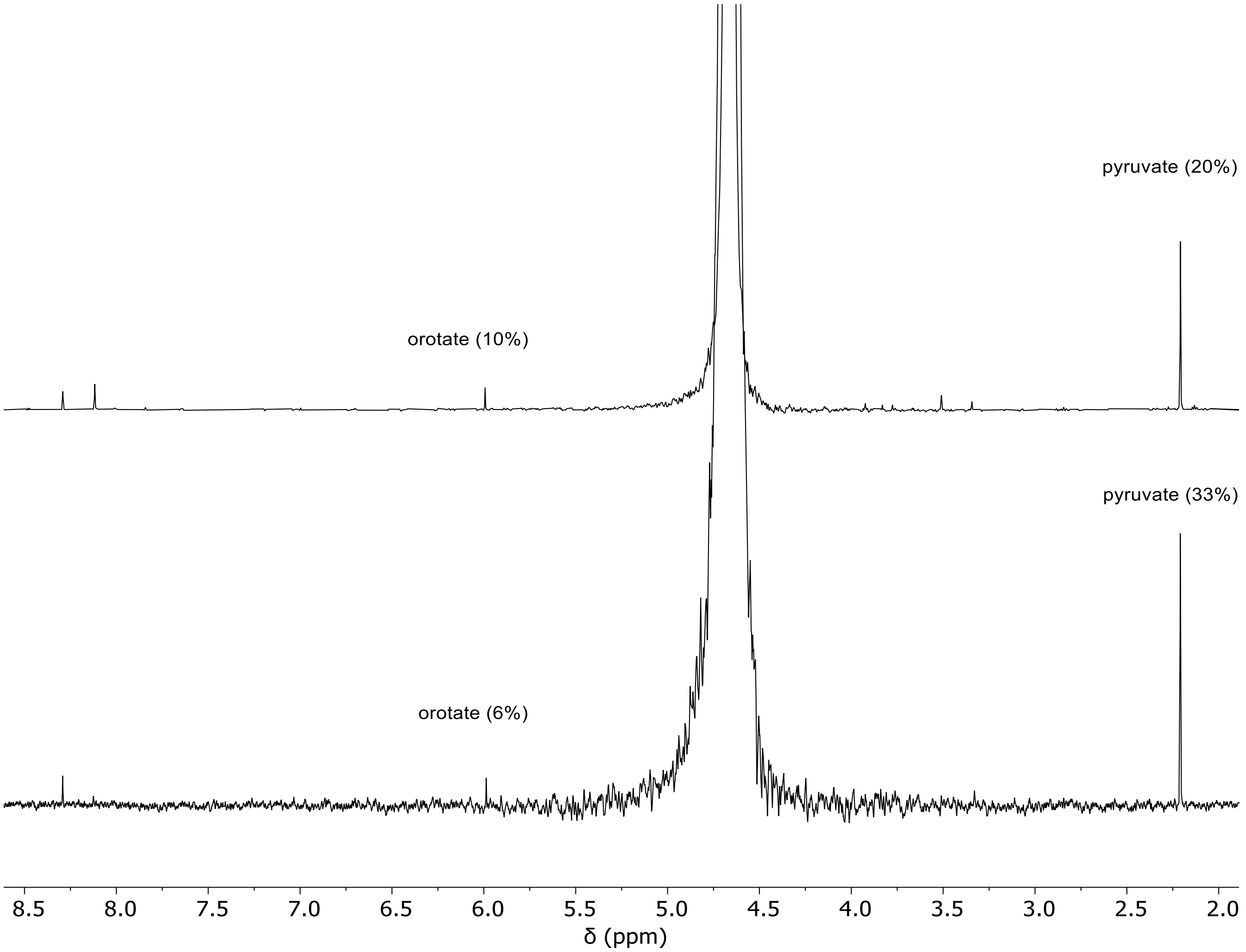

Figure S8. Effect of hydantoin concentration on the production of pyruvate and orotate. ^{1}H NMR (in D_2O) of a reaction aliquot from (A) 90 mM, (B) 15 mM hydantoin in 1.0 M $NaHCO_3$, pH 8.2, with 1.5 eq. of sodium glyoxylate, stirred at 80 °C for 3 days.

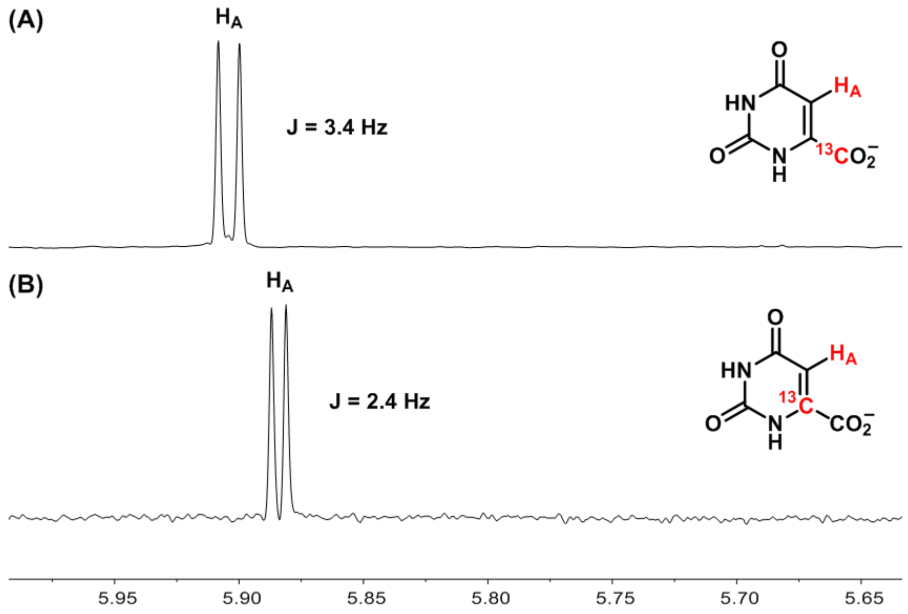

Figure S9. ^{1}H NMR spectra in D_2O showing the coupling constants for **(A)** (7-^{13}C)orotate and **(B)** (6-^{13}C)orotate.

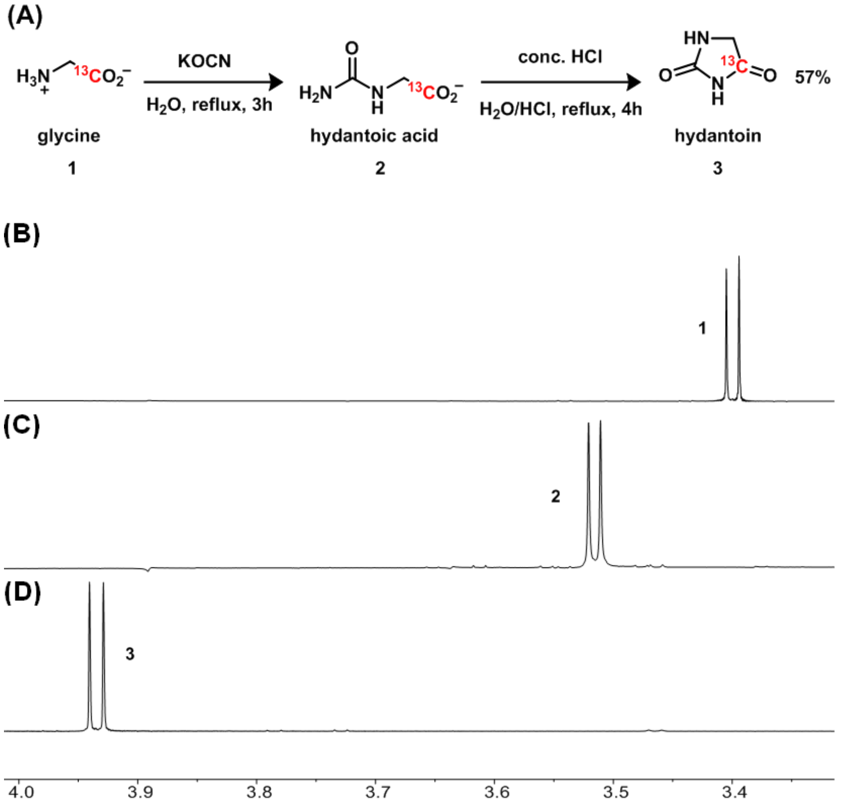

Figure S10. (A) Reaction scheme for (4-^{13}C)hydantoin synthesis. ^{1}H NMR spectra in D_2O of **(B)** (1-^{13}C)glycine, **(C)** (1-^{13}C)hydantoic acid, **(D)** and (4-^{13}C)hydantoin.

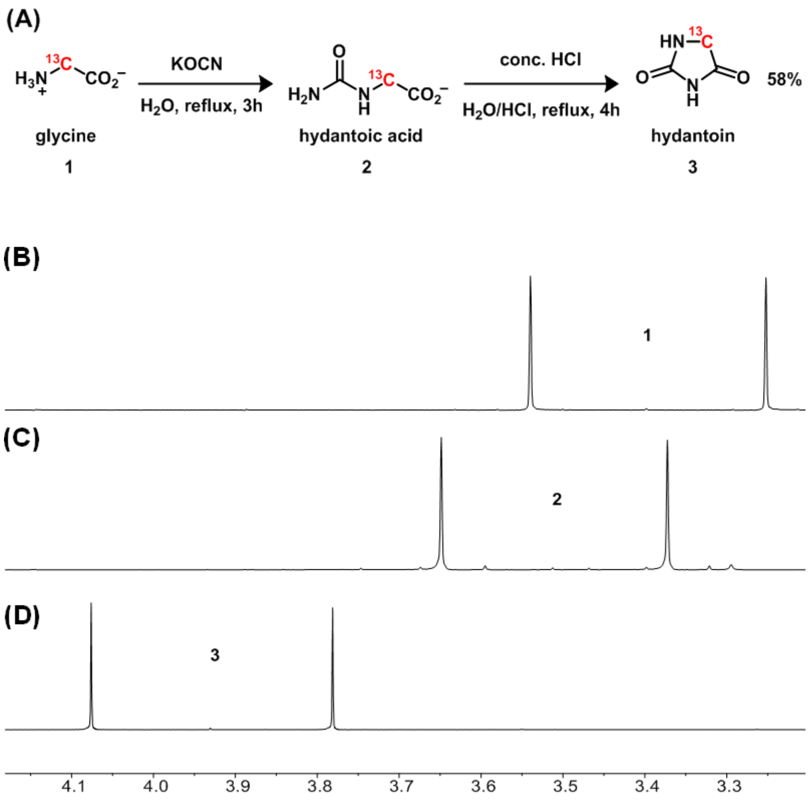

Figure S11. (A) Reaction scheme for (5-^{13}C)hydantoin synthesis. ^{1}H NMR spectra in D_2O of **(B)** (2-^{13}C)glycine, **(C)** (2-^{13}C)hydantoic acid, **(D)** and (5-^{13}C)hydantoin.

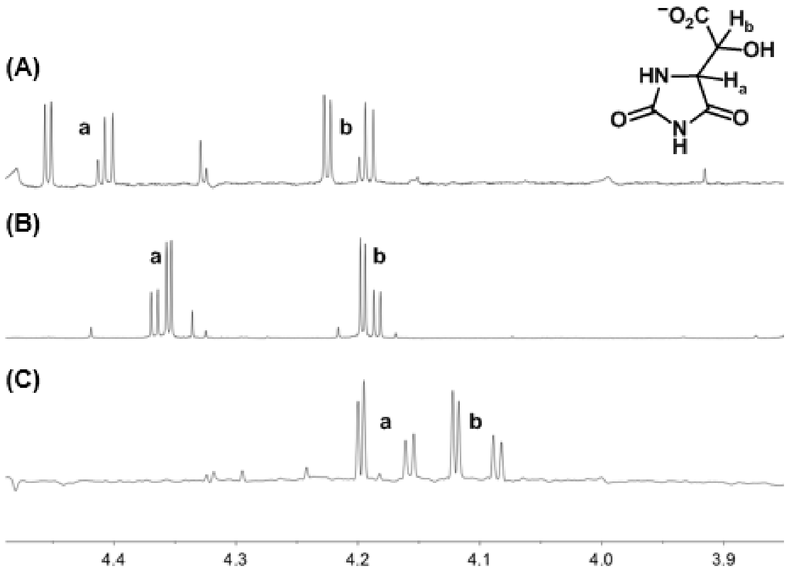

Figure S12. Aldol addition of hydantoin and glyoxylate to produce intermediate I occurs at a range of pH values. **(A)** ^{1}H NMR (in D_2O) of a reaction aliquot from 90 mM hydantoin with 1.5 eq. of glyoxylic acid, stirred at 60 °C for 2 hours in 1.0 M aq. phosphate buffer, pH 5.5, (**B**) for 1 hour in 1.0 M aq. $NaHCO_3$, pH 8.2, and **(C)** for 4 hours in aq. sat. Na_2CO_3, pH 11.

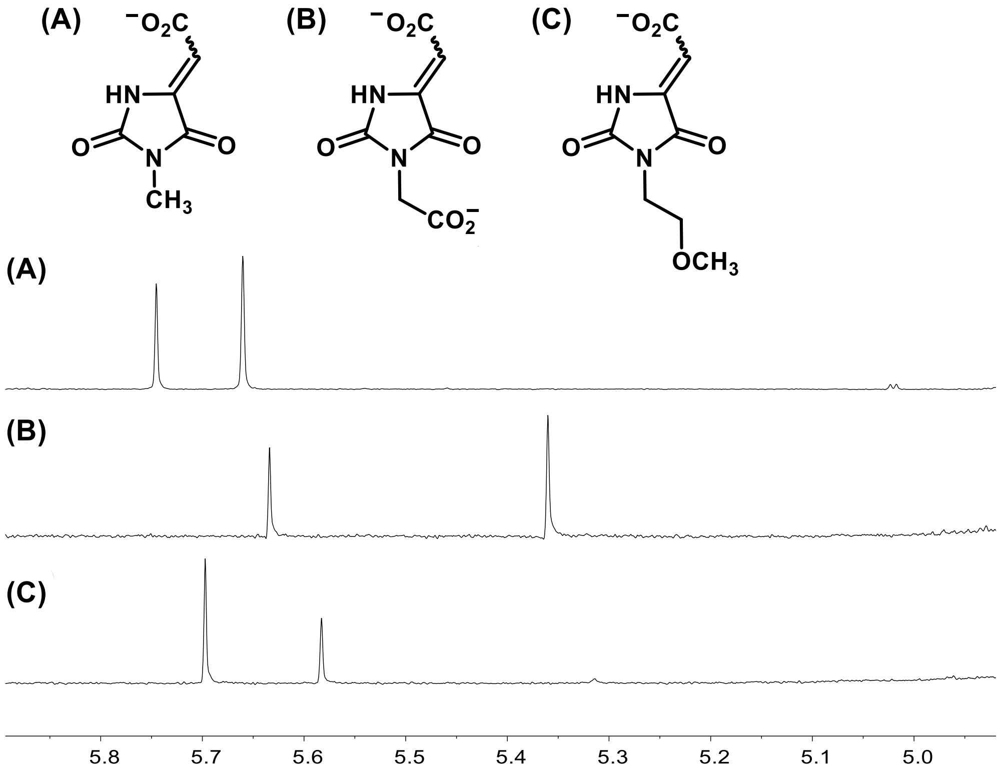

Figure S13. Orotate nucleoside synthesis halts at (*E*,*Z*)-5-carboxymethylidine-hydantoin intermediates. ^{1}H NMRs (in D_2O) of reaction aliquots from: **(A)** 90 mM 1-methylhydantoin, **(B)** 90 mM hydantoin-1-acetic acid, and **(C)** 90 mM 1-methyoxyethylhydantoin in 1.0 M $NaHCO_3$, pH 8.2, with 1.5 eq. of glyoxylic acid, stirred at 60 °C for 8 days.

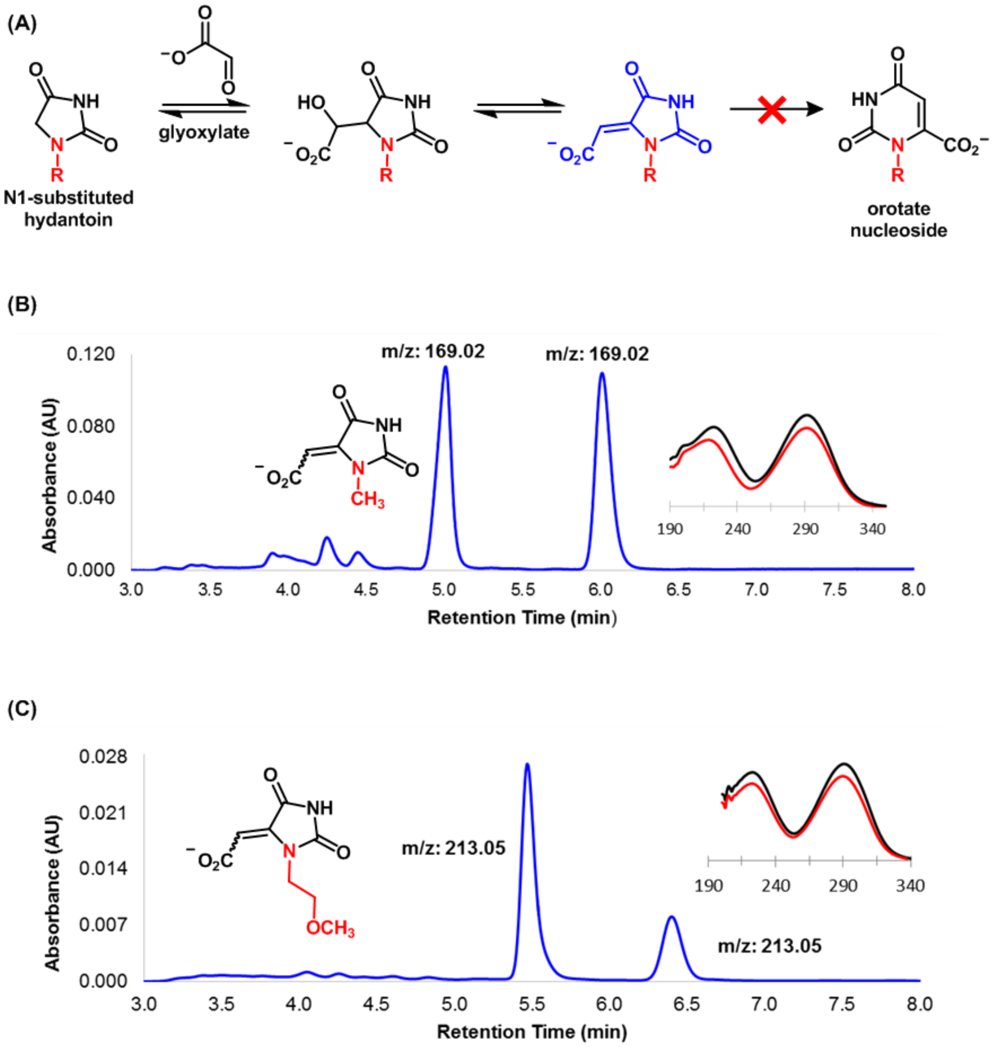

Figure S14. *E* and *Z* 5-carboxymethylidine-hydantoins. **(A)** Hypothetical orotate nucleoside generation from *N*-1 substituted hydantoin and glyoxylate. **(B)** LCMS chromatogram from a reaction aliquot of 90 mM 1-methylhydantoin in 1.0 M $NaHCO_3$, pH 8.2, with 1.5 eq. of glyoxylic acid, stirred at 60 °C for 8 days. **(C)** LCMS chromatogram from a reaction aliquot of 90 mM 1-methyoxyethylhydantoin in 1.0 M $NaHCO_3$, pH 8.2, with 1.5 eq. of glyoxylic acid, stirred at 60 °C for 8 days.

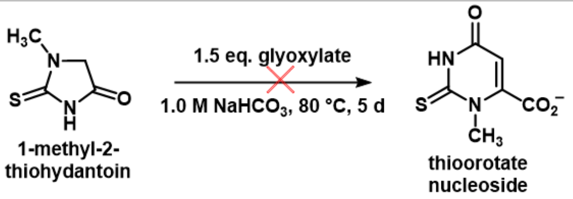

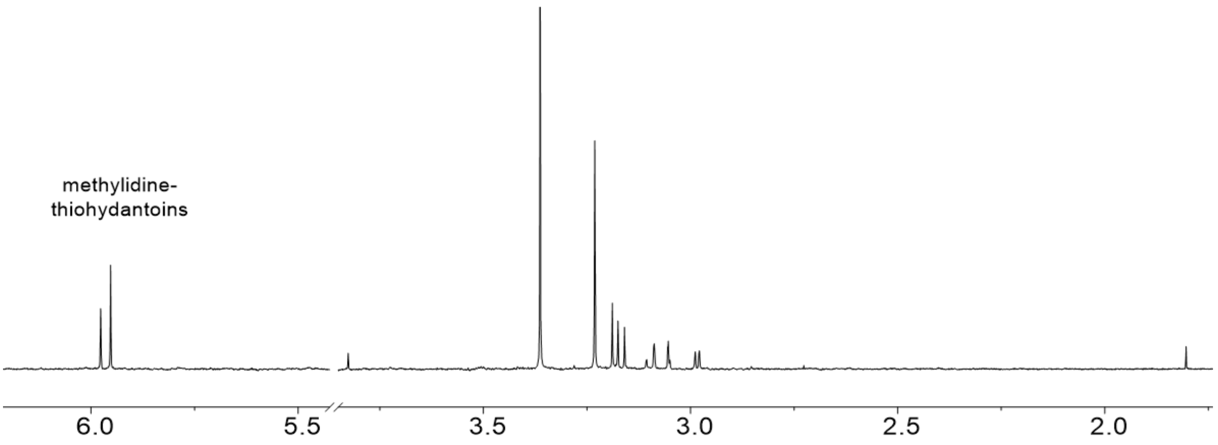

Figure S15. Attempt to synthesize 1-methyl-2-thioorotate nucleoside derivative from 1-methyl-2-thiohydantoin.^{1}H NMR spectra in D_2O of a reaction aliquot from 90 mM 1-methyl-2-thiohydantoin in 1.0 M $NaHCO_3$, pH 8.2 with 1.5 eq. of glyoxylic acid, stirred at 80 °C for 24 h.

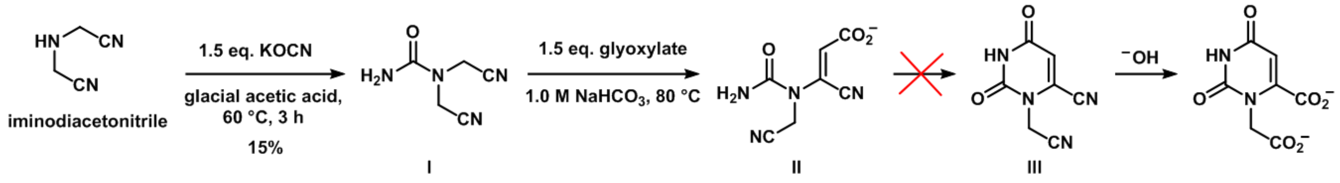

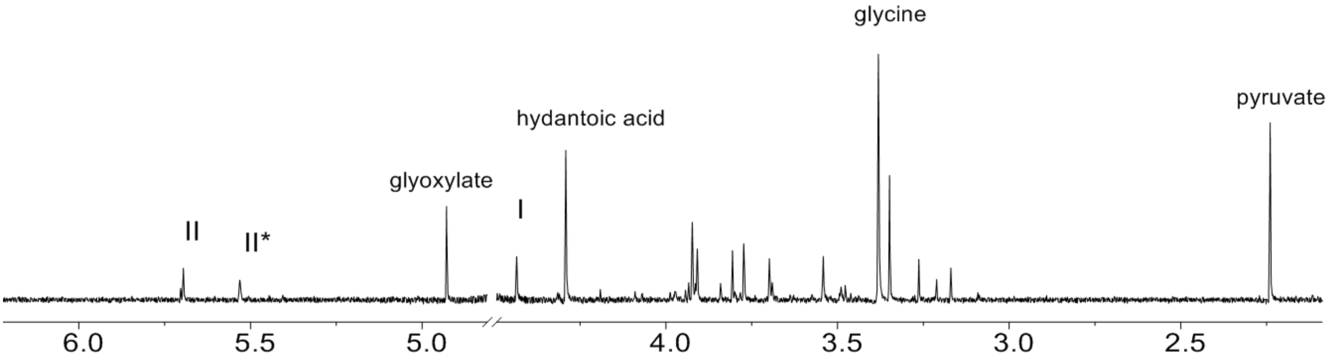

Figure S16. Attempted synthesis of 1-carboxymethyl orotate nucleoside derivative. ^{1}H NMR spectra in D_2O of a reaction aliquot from 90 mM intermediate I in 1.0 M $NaHCO_3$, pH 8.2 with 1.5 eq. of glyoxylic acid, stirred at 80 °C for 3 days.

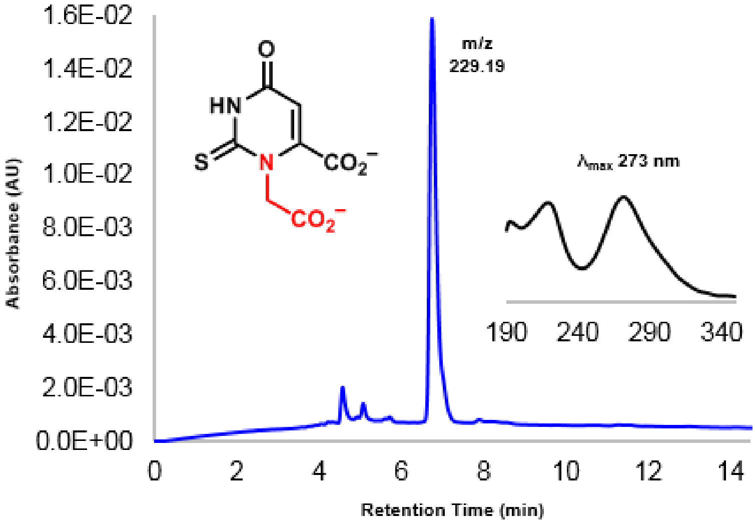

Figure S17. Synthesis of 1-carboxymethyl-2-thioorotate. LCMS chromatogram at 270 nm of a reaction aliquot from 90 mM 1-cyanomethyl-1-(carboxymethyl)thiourea in 1.0 M $NaHCO_3$, pH 8.2 with 1.5 eq. of glyoxylic acid, stirred at 80 °C for 3 days.

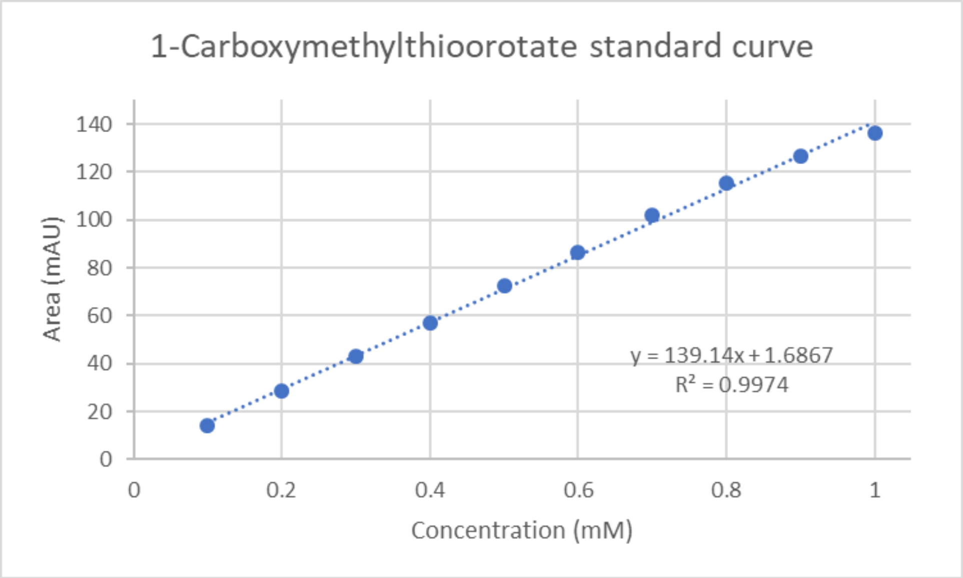

Figure S18. HPLC standard curve generated for 1-carboxymethyl-2-thioorotate (which was used for yield calculation). Area of the crude material (5 mg/mL solution) by using the same HPLC conditions = 36.3934 mAU; Calculated concentration of the crude material solution (5 mg/mL) = 0.249 mM; Total amount of the crude material obtained from the reaction mixture (started from 1.5 mmols) = 3.57g; Yield of the reaction = 11.9%. HPLC used for this experiment is Thermofisher Ultimate 3000 (see experimental procedures for more details).
